# Measurement bias detection with Kronecker product restricted models for multivariate longitudinal data: an illustration with health-related quality of life data from thirteen measurement occasions

**DOI:** 10.3389/fpsyg.2014.01022

**Published:** 2014-09-23

**Authors:** Mathilde G. E. Verdam, Frans J. Oort

**Affiliations:** ^1^Department of Medical Psychology, Academic Medical Centre, University of AmsterdamAmsterdam, Netherlands; ^2^Faculty of Social and Behavioral Sciences, Research Institute Child Development and Education, University of AmsterdamAmsterdam, Netherlands

**Keywords:** Kronecker product, multivariate longitudinal data, measurement invariance, structural equation modeling (SEM), longitudinal three-mode model (L3MM), health-related quality of life (HRQoL)

## Abstract

**Highlights:**

- Application of Kronecker product to construct parsimonious structural equation models for multivariate longitudinal data.
- A method for the investigation of measurement bias with Kronecker product restricted models.
- Application of these methods to health-related quality of life data from bone metastasis patients, collected at 13 consecutive measurement occasions.
- The use of curves to facilitate substantive interpretation of apparent measurement bias.
- Assessment of change in common factor means, after accounting for apparent measurement bias.

Longitudinal measurement invariance is usually investigated with a longitudinal factor model (LFM). However, with multiple measurement occasions, the number of parameters to be estimated increases with a multiple of the number of measurement occasions. To guard against too low ratios of numbers of subjects and numbers of parameters, we can use Kronecker product restrictions to model the multivariate longitudinal structure of the data. These restrictions can be imposed on all parameter matrices, including measurement invariance restrictions on factor loadings and intercepts. The resulting models are parsimonious and have attractive interpretation, but require different methods for the investigation of measurement bias. Specifically, additional parameter matrices are introduced to accommodate possible violations of measurement invariance. These additional matrices consist of measurement bias parameters that are either fixed at zero or free to be estimated. In cases of measurement bias, it is also possible to model the bias over time, e.g., with linear or non-linear curves. Measurement bias detection with Kronecker product restricted models will be illustrated with multivariate longitudinal data from 682 bone metastasis patients whose health-related quality of life (HRQL) was measured at 13 consecutive weeks.

A valid assessment of change requires that the meaning of the construct stays the same across measurement occasions (Meredith, 1993). Longitudinal measurement invariance is usually investigated with the longitudinal factor model (LFM). When *R* latent variables are measured with *K* observed variables at *J* measurement occasion, the mean, and covariance structures are given by:

(1) μ=τ+Λκ,

and:

(2) $$\Sigma =\Lambda \Phi \Lambda^{\prime }+\Theta ,$$

where **τ** is a *JK*-vector of intercepts, **Λ** is a *JK* × *JR* matrix of common factor loadings, **κ** is a *JR*-vector of common factor means, **Φ** is a *JR* × *JR* symmetric matrix containing the variances and covariances of the common factors, and **Θ** is a *JK* × *JK* symmetric matrix containing the variances and covariances of the residual factors. Although the LFM can be used to model multiple measurement occasions, these models become progressively large and unmanageable when the number of occasions increases.

One solution to this problem is the imposition of Kronecker product restrictions that profit from the three-mode structure of multivariate longitudinal data (Oort, 2001). The modes refer to the variables, the measurement occasions and the subjects, and the resulting longitudinal three-mode models (L3MMs) are more parsimonious and have attractive interpretation. For example, Kronecker product restrictions can be imposed on factor loadings and intercepts to comply with measurement invariance, using:

(3) $$\Lambda =I⊗\Lambda_{0},$$

and:

(4) $$\tau =u⊗\tau_{0},$$

where **Λ**_0_ is a *K* × *R* matrix of invariant common factor loadings, **τ**_0_ is a *K* × 1 vector of invariant intercepts, **I** is a *J* × *J* identity matrix, **u** is a *J* × 1 vector of ones, and the symbol ⊗ denotes the Kronecker product. These restrictions imply that factor loadings **Λ**_0_ and intercepts τ_0_ apply to all measurement occasions. Although Kronecker product restrictions are convenient to model measurement invariance, they require special methods for the investigation of violations of measurement invariance (i.e., measurement bias).

Specifically, to detect measurement bias in Kronecker product restricted models, we introduce additional matrices **A** and **B** to accommodate possible violations of measurement invariance, using:

(5) $$\Lambda =I⊗\Lambda_{0}+A,$$

and:

(6) $$\tau =u⊗\tau_{0}+B.$$

These additional matrices consist of measurement bias parameters that are either fixed at zero or free to be estimated. This method thus enables the detection of measurement bias in individual parameters of **Λ** and **τ**. In this way, it is possible to establish partial measurement invariance (Byrne et al., 1989). Moreover, in cases of measurement bias, it is also possible to model the bias over time, e.g., with linear or non-linear curves, which can facilitate interpretation.

The aim of the present paper is to illustrate the detection of measurement bias with Kronecker product restricted models using multivariate longitudinal data from 682 bone metastasis patients whose health-related quality of life (HRQL) was measured in 13 consecutive weeks.

## Methods

Patients with painful bone metastases from a solid tumor were enrolled from 17 radiotherapy institutes in The Netherlands. Patients were randomized to receive radiotherapy of a single fraction vs. multiple fractions as palliative treatment for pain. Inclusion criteria were having one or more painful bone metastases treatable in one target volume and having a pain score of at least 2 on an 11-point scale from 0 (no pain at all) to 10 (worst imaginable pain) at time of admission to the radiotherapy. Exclusion criteria were having metastases of malignant melanoma or renal cell carcinoma, having metastases in the cervical spine, having previously been irradiated for the same metastases, or having a pathological fracture that needed surgical fixation or a spinal cord compression. Side effects from radiation therapy vary depending on the part of the body being treated, and may include skin changes (dryness, itching, peeling, or blistering), fatigue, loss of appetite, hair loss, diarrhea, nausea, and vomiting. Most of these side effects go away within a few weeks after radiation therapy.

HRQL questionnaires were administered at 13 measurement occasions, before (T0) and every week after treatment with radiotherapy (T1 through T12). These data are a subset of data from the Dutch Bone Metastasis Study (Steenland et al., 1999; Van der Linden et al., 2004). For the current study only patients who survived at least 13 weeks from the start of treatment were included, which resulted in a total sample size of 682 patients (354 women). Patients' primary tumor was either breast cancer (*n* = 321), prostate cancer (*n* = 181), lung cancer (*n* = 106), or other (*n* = 74). Ages ranged from 33 to 90, with a mean of 64.2 (standard deviation 11.5).

Treatment progression, therapeutic effects and/or side effects may influence patients' health status. In the area of HRQL a theoretical framework of measurement bias has been developed which describes how changes in patients' health status may prompt behavioral, cognitive, and affective processes that affect patients' response tendencies (Sprangers and Schwartz, 1999). Therefore, it seems worthwhile to investigate measurement bias in our sample of bone metastases patients.

### Measures

HRQL was assessed with multiple questionnaires (for more information see Verdam et al., submitted). Forty-five Items were grouped using confirmatory factor analyses and substantive considerations to compute eight health-indicators: physical functioning (PF; 4 items), mobility (MB; 5 items), social functioning (SF; 2 items), depression (DP; 8 items), listlessness (LS; 6 items), pain (PA; 4 items), sickness (SI; 6 items), and treatment related symptoms (SY; 11 items). All scale scores were calculated as mean item scores, ranging from 1 to 4, with higher scores indicating more symptoms or dysfunctioning.

Intermittent missing item- and scale scores were imputed using expectation-maximization. Per assessment, 29–35% of respondents showed missing item scores and 1–3% of respondents showed intermittent missing scale scores. Cronbach's alpha coefficients indicated moderate to good internal consistency reliability (PF, alpha = 0.93; MB, alpha = 0.91; SF, alpha = 0.80; DP, alpha = 0.94; LS, alpha = 0.72; SI, alpha = 0.74; PA alpha = 0.74; SY, alpha = 0.69).

### Structural equation modeling

Structural equation models were fitted to the means, variances and covariances of the eight observed health indicators using OpenMx (Boker et al., 2011). OpenMx syntax is available in Appendix I^1^. To achieve identification of all model parameters, scales and origins of the common factors were established by fixing the factor means at zero and the factor variances at one. When factor loadings and intercepts were constrained to be equal across occasion, only first occasion factor means and variances were fixed. Model parameters of the additional matrices **A** and **B** can be freely estimated, with the restriction that the computed matrices of factor loadings and intercepts do not violate the general guidelines for identification as described above. Identification of model parameters of matrices that feature in the Kronecker product restriction imposed on residual factor variances and covariances was achieved by using the guidelines described by Oort (2001).

#### Detection of measurement bias

The structural equation modeling procedure for the detection of measurement bias included the following steps: (1) establishing an appropriate measurement model, (2) fitting a model of measurement invariance, (3) detection of measurement bias, (4) modeling the bias that was detected, and (5) assessment of change.

***Step 1: measurement model***. The *Measurement Model* was established on the basis of results of exploratory factor analyses and substantive considerations. To take into account the multivariate longitudinal structure of the data, the longitudinal three-mode model (L3MM; Oort, 2001) was applied. To reduce the complexity of the model (i.e., the number of parameter estimates) Kronecker product restrictions were imposed on residual variances and covariances, using **Θ** = **Θ**_T_ ⊗ **Θ**_V_. This restriction entails that the matrix of residual variances and covariances (**Θ**) is estimated indirectly by using a symmetric matrix that contains the relationships between measurement occasions (**Θ**_T_, of dimensions 13 × 13; with **Θ**_T(1,1)_ = 1 for identification purposes) and a diagonal matrix that contains the residual variances of only one measurement occasion (**Θ**_V_, of dimension 8 × 8). This implies that the changes in residual factor variances and covariances across occasions are proportionate for all residual factors (for more details see Verdam et al., submitted). The *Measurement Model* has no equality constraints across occasions.

***Step 2: measurement invariance model***. The assumption of longitudinal measurement invariance entails that factor loadings and intercepts are constrained to be equal across occasions. These restrictions were imposed using the Kronecker product with Equations (3) and (4), yielding the *Measurement Invariance Model*. To test the assumption of measurement invariance the model fit of the more restricted model can be compared to the model fit of the model with no equality constraints across occasions. When there is no significant deterioration in model fit, the assumption of measurement invariance can be retained.

***Step 3: partial measurement invariance model***. Detection of measurement bias was done using a step-by-step modification of the *Measurement Invariance Model*, to yield the *Partial Measurement Invariance Model* which included all occurrences of measurement bias. Measurement bias was operationalized as differences across measurement occasions in parameter estimates of factor loadings or intercepts. An iterative procedure was used, where each invariant factor loading and intercept was investigated one-by-one. Using Equations (5) and (6) all measurement bias parameters across occasions that were associated with one invariant parameter were freely estimated. The violations of measurement invariance that yielded the largest improvement in model fit were incorporated in the model. To test whether partial measurement invariance is tenable the model fit of this model can be compared to the model fit of the model with no equality constraints across measurement occasions. When there is no significant deterioration in model fit, the assumption of partial measurement invariance can be retained. The final model, the *Partial Measurement Invariance Model*, thus includes measurement invariance restrictions on most, but not all, factor loading and intercept parameters.

***Step 4: modeling occurrences of measurement bias***. In case of measurement bias, the bias was modeled using linear or non-linear curves. The measurement bias parameters were modeled as a function of the time of measurement (using a time-coding), yielding estimates of intercept- and slope-parameters that describe the trend of the bias. When the model fit of the more restricted model did not significantly deteriorate compared to the model fit of the model with freely estimated measurement bias parameters, we retained the model which describes the bias using a linear or non-linear curve. Interpretation of parameter estimates provides insight in the trend of the bias that was detected.

***Step 5: assessment of change***. Change in the common factor means was assessed in the model where all measurement biases were taken into account. A test of invariance was used to investigate differences in common factor means across occasions. To evaluate the impact of measurement bias on the assessment of change, we inspected the trajectories of common factor means, before and after taking into account measurement bias.

#### Evaluation of model fit

To evaluate goodness-of-fit the chi-square test of exact fit (CHISQ) was used, where a significant chi-square indicates a significant difference between model and data. However, in the practice of structural equation modeling, exact fit is rare, and with large sample sizes and large numbers of degrees of freedom the chi square test generally turns out to be significant. Therefore, we also considered alternative measures of fit. The root mean square error of approximation (RMSEA; Steiger and Lind, 1980; Steiger, 1990) was used as a measure of approximate fit, where RMSEA values below 0.05 indicate “close” approximate fit and values below 0.08 indicate “reasonable” approximate fit (Browne and Cudeck, 1992). Additionally, the expected cross-validation index (ECVI; Browne and Cudeck, 1989) was used to compare different models for the same data, where the model with the smallest ECVI indicates the model with the best fit. For both the RMSEA and ECVI, 95% confidence intervals were calculated using the program NIESEM (Dudgeon, 2003).

To evaluate differences between hierarchically related models the chi-square difference test (CHISQ_*diff*_) was used, where a significant chi-square difference indicates a significant difference in model-fit. The chi square difference test applied to hierarchically nested models has essentially the same strengths and weaknesses as the chi square test applied to a single model. Therefore, we additionally considered the ECVI difference test (ECVI_*diff*_), where the deterioration in model fit of the more restricted model is significant when the value of the ECVI difference is significantly larger than zero.

## Results

### Measurement model

**Figure 1:**
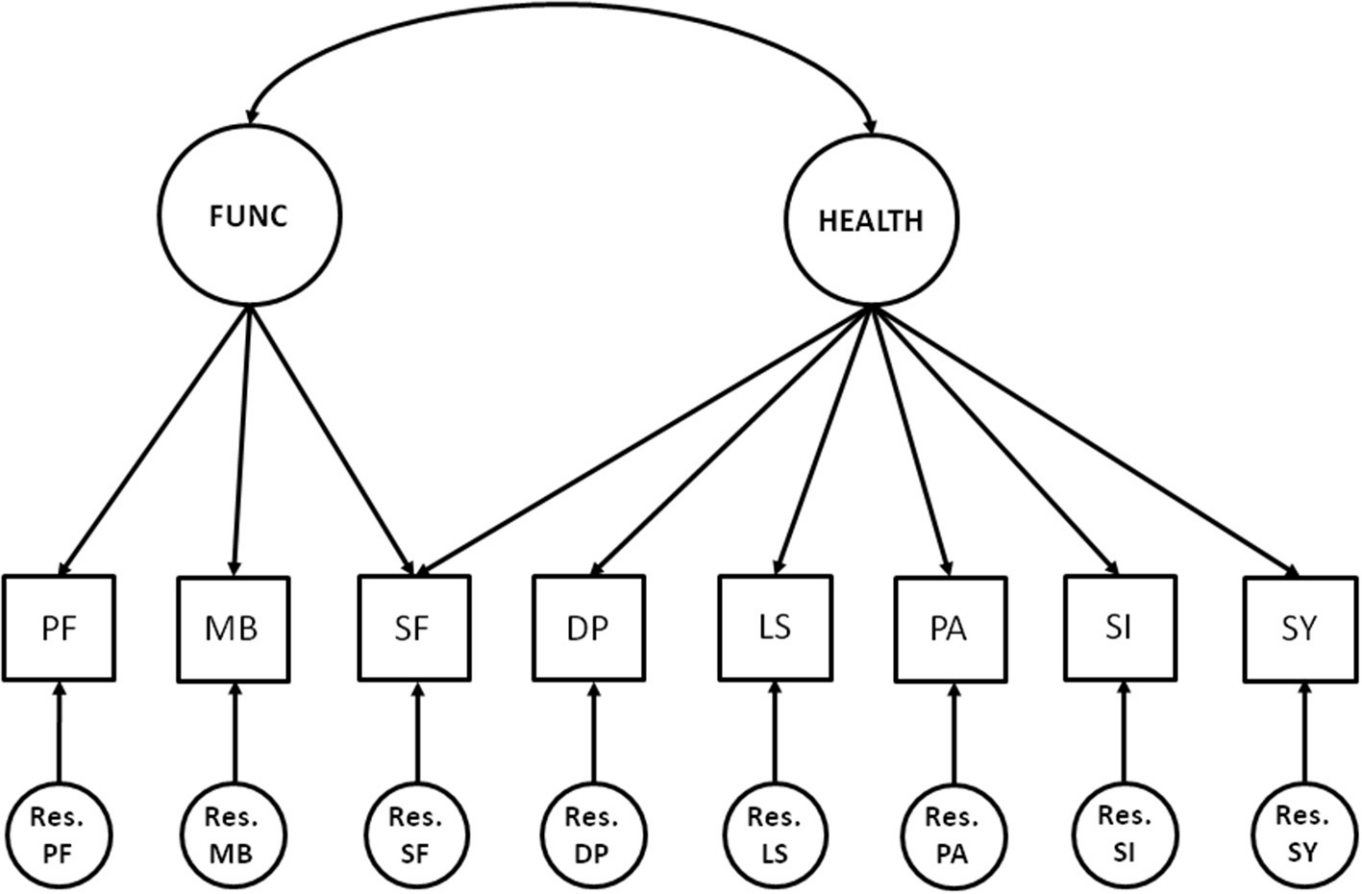
**The measurement model**. Circles represent latent variables (common and residual factors) and squares represent observed variables (the scale scores). FUNC, functional limitations; HEALTH, health impairments; PF, physical functioning; MB, mobility; SF, social functioning; DP, depression; LS, listlessness; PA, pain; SI, sickness; SY, treatment related symptoms; and Res., Residual factors.

Eight health-indicators were modeled to be reflective of two common factors: functional limitations and health impairments (see Figure 1). Functional limitations was measured by three observed variables, health impairments was measured by six observed variables, with one observed variable in common. The squares represent observed variables (scale scores), the circles on the top represent the common factors, and the circles on the bottom represent residual factors.

Classification of the common factors was based on the International Classification of Functioning, Disability and Health (World Health Organization, 2002) that provides a framework for the description of health and health-related states. In this framework, the term functioning refers to all body functions, activities and participation, while disability refers to impairments, activity limitations and participation restrictions. These concepts are reflected in the two common factors functional limitations (e.g., limitations of bodily functioning) and health impairments (e.g., health restrictions or symptoms). As social functioning is also considered to be an important factor of HRQL, this scale was added to the measurement and modeled to be influenced by both functional limitations and health impairments (which agrees with participation being a factor of both functioning and disability in the WHO framework).

The *Measurement Model* yielded a chi-square test of exact fit that was significant but the RMSEA measure indicated close fit (see Model 1, Table 1).

**Table 1:** **Goodness of overall fit of models in the four-step measurement bias detection procedure**.

**Model**	**Description**	**DF**	**CHISQ**	**RMSEA [95% CI]**	**ECVI [95% CI]**
Model 1	Measurement model	4920	9094.7	0.035 [0.034;0.036]	15.59 [15.11; 16.09]
Model 2	Measurement invariance model	5076	9829.9	0.037 [0.036;0.038]	16.13 [15.62; 16.66]
Model 3	Partial measurement invariance model	5040	9318.2	0.035 [0.034;0.037]	15.50 [15.01; 16.01]
Model 4	Curves partial measurement invariance model	5070	9380.8	0.035 [0.034;0.037]	15.49 [15.00; 16.00]

*n = 682*.

### Detection of measurement bias

To test the assumption of longitudinal measurement invariance, factor loadings and intercepts were held invariant across occasions using the Kronecker product restriction. The overall fit of the *Measurement Invariance Model* showed reasonable fit (RMSEA = 0.037, see Table 1), but comparison with the fit of the model with no across occasions equality constraints showed a significant deterioration in fit [CHISQ_*diff*_ (156) = 735.2, *p* < 0.001; ECVI_*diff*_ = 0.54, 95% CI: 0.39–0.71]. This indicates a violation of measurement invariance.

To investigate which of the equality constraints across occasions on factor loadings and intercepts were not tenable, an iterative measurement bias detection approach was used. Step by step modification of the *Measurement Invariance Model* yielded the *Partial Measurement Invariance Model*, which showed three cases of measurement bias. Each of the measurement biases that was detected will be explained in more detail below. The fit of the *Partial Measurement Invariance Model* was good (RMSEA = 0.035, see Table 1), and significantly better than the fit of the *Measurement Invariance Model* [CHISQ_*diff*_ (36) = 511.7, *p* < 0.001; ECVI_*diff*_ = 0.63, 95% CI: 0.50–0.77]. Moreover, comparison with the *Measurement Model* showed that although there was still a significant difference in fit according to the chi-square difference test, comparison of approximate fit using the ECVI difference test indicated that the models can be considered approximately equivalent [CHISQ_*diff*_ (120) = 223.5, *p* < 0.001; ECVI_*diff*_ = −0.09]. Therefore, the *Partial Measurement Invariance Model* was retained. All invariant parameters of **Λ**_0_ and τ_0_, and the measurement bias parameters of the three cases of bias, are given in Tables 2,3, respectively.

**Table 2:** **Measurement invariant parameter estimates of the Partial Measurement Invariance Model**.

	**PF**	**MB**	**SF**	**DP**	**LS**	**PA**	**SI**	**SY**
**INTERCEPTS (τ_0_)**								
	3.03	2.12	2.25	1.98	2.29	**Bias**	**Bias**	1.46
**FACTOR LOADINGS (Λ_0_)**								
FUNC	0.90	0.70	0.29					
HEALTH			0.27	0.39	0.43	0.35	**Bias**	0.19

*N = 682; parameter estimates are unstandardized*.

**Table 3:** **Measurement bias parameter estimates of the Partial Measurement Invariance Model**.

**T0**	**T1**	**T2**	**T3**	**T4**	**T5**	**T6**	**T7**	**T8**	**T9**	**T10**	**T11**	**T12**
**INTERCEPT “PAIN”**													
2.56	2.41	2.33	2.27	2.22	2.21	2.21	2.21	2.21	2.21	2.21	2.21	2.21
**INTERCEPT “SICKNESS”**													
1.37	1.49	1.56	1.56	1.52	1.49	1.47	1.46	1.46	1.46	1.45	1.43	1.44
**FACTOR LOADING “SICKNESS”**													
0.28	0.35	0.40	0.41	0.37	0.34	0.34	0.33	0.33	0.33	0.32	0.29	0.31

*N = 682; parameter estimates are unstandardized*.

#### Measurement bias intercept “pain”

The first bias that was detected was a measurement bias of the indicator “pain.” The model where the intercept of the indicator “pain” was freely estimated across occasions yielded the largest improvement in model fit [CHISQ_*diff*_ (12) = 287.7, *p* < 0.001; ECVI_*diff*_ = 0.38, 95% CI: 0.28–0.49]. Inspection of the measurement bias parameters shows that the estimate of the intercept decreases over the first five measurement occasions and stabilizes around the sixth measurement occasion (see Table 3). This indicates that, given equal health impairments, patients report decreasing pain over the first 4 weeks after treatment, after which they report stable pain over time.

**Figure 2:**
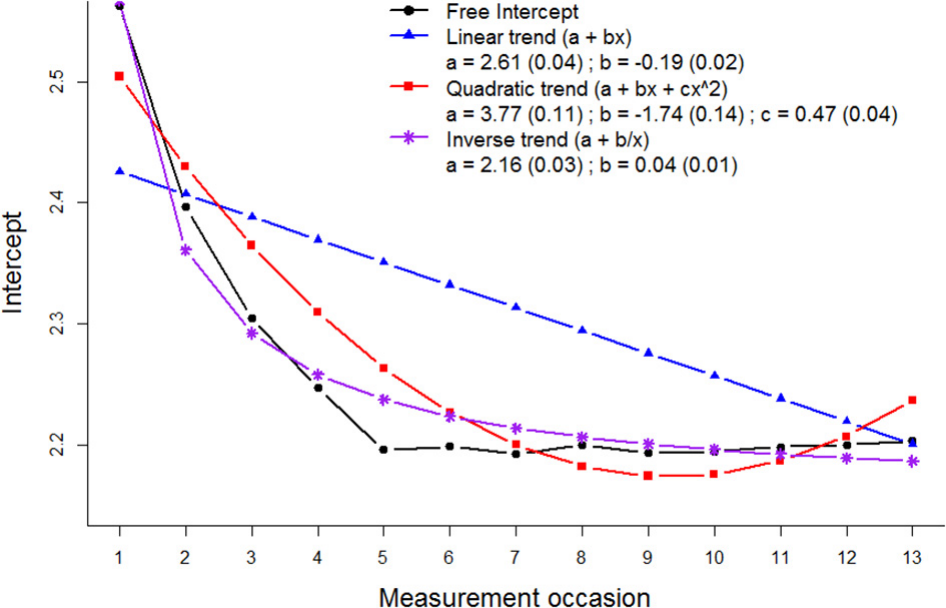
**Curves fitted to the measurement bias parameters of the intercept “pain.”** The black line (circles) represents measurement bias parameter estimates when they are freely estimated across occasions, the blue line (triangles) when they are modeled using a linear curve, the red line (squares) when they are modeled using a quadratic curve, and the purple line (stars) when they are modeled using an inverse curve.

To get more insight in the trend of this bias, the measurement bias parameters were modeled as a function of the time of measurement. First, a linear curve was fitted to the bias. This model yielded an intercept and slope parameter that can give insight in the trend of the bias across occasions (see Figure 2), but the model did not show a good fit to the data [CHISQ_*diff*_ (11) = 189.9, *p* < 0.001; ECVI_*diff*_ = 0.24, 95% CI: 0.16–0.33]. In addition, a selection of non-linear curves was fitted to the measurement bias parameters (see Figure 2) of which the quadratic curve showed significant deterioration in fit [CHISQ_*diff*_ (10) = 61.0, *p* < 0.001; ECVI_*diff*_ = 0.05, 95% CI: 0.02–0.11], but the inverse curve showed equivalent fit to the model with free intercepts [CHISQ_*diff*_ (10) = 18.7, *p* = 0.044; ECVI_*diff*_ = −0.01]. The slope parameter gives an indication of the steepness and direction of the measurement bias for the first five measurement occasions.

#### Measurement bias intercept “sickness”

The second step of the measurement bias detection procedure showed that the equality constraint on the intercept of the indicator “sickness” across occasions was not tenable [CHISQ_*diff*_ (12) = 141.9, *p* < 0.001; ECVI_*diff*_ = 0.17, 95% CI: 0.10–0.25]. Inspection of the measurement bias parameters shows that the intercept of the indicator “sickness” increases over the first four measurement occasions, after which it decreases and stabilizes around the seventh measurement (see Table 3). Thus, given equal health impairments, patients report more sickness in the first 3 weeks after treatment, then report less sickness, and after the sixth week after treatment report a stable, above baseline level of sickness.

**Figure 3:**
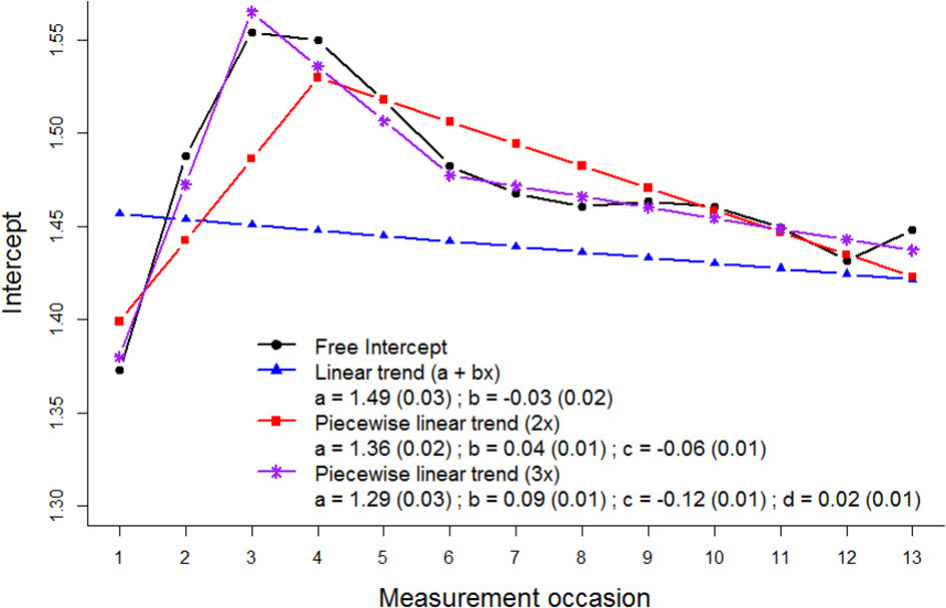
**Curves fitted to the measurement bias parameters of the intercept “sickness.”** The black line (circles) represents measurement bias parameter estimates when they are freely estimated across occasions, the blue line (triangles) when they are modeled using a linear curve, the red line (squares) when they are modeled using two piece-wise linear curves, and the purple line (stars) when they are modeled using three piece-wise linear curves.

A model with a linear curve was fitted to the data, which yielded a non-significant slope parameter estimate (see Figure 3), and showed significant deterioration in fit compared to the model with free intercepts [CHISQ_*diff*_ (11) = 138.2, *p* < 0.001; ECVI_*diff*_ = 0.16, 95% CI: 0.10–0.25]. As it can be seen from the data that different parts of the trajectory of the intercept follow different trends (i.e., first an increase and then a decrease across measurement occasions), we modeled these trajectories in the bias using piece-wise curves. Piece-wise curves were modeled using additional time coding that applied to only part of the trajectory. In this example, linear piece-wise curves were fitted to the measurement bias parameters of “sickness” (see Figure 3), where the model with two piece-wise curves did not show a good fit to the data [CHISQ_*diff*_ (10) = 64.7, *p* < 0.001; ECVI_*diff*_ = 0.06, 95% CI: 0.02–0.12], but the model with three piece-wise curves showed equivalent fit to the model with free intercepts [CHISQ_*diff*_ (10) = 11.0, *p* = 0.274; ECVI_*diff*_ = −0.02]. The slope parameters give an indication of the steepness and direction of the measurement bias for the first three measurement occasions, and the deviations from this trend for the fourth to sixth measurement occasions, and the seventh to thirteenth measurement occasions (see Figure 3).

#### Measurement bias factor loading “sickness”

The third bias that was detected was a measurement bias of the indicator “sickness,” as freeing the equality constraint on the factor loading across occasions yielded the largest improvement in model fit [CHISQ_*diff*_ (12) = 82.0, *p* < 0.001; ECVI_*diff*_ = 0.08, 95% CI: 0.03–0.14]. Inspection of the measurement bias parameters shows that the factor loading increases over the first four measurement occasions, after which it decreases again toward baseline level, although it shows a somewhat fluctuating pattern (see Table 3). Thus, sickness becomes more important for patients' health impairments in the first 3 weeks after treatment, but then its importance decreases again toward baseline level.

**Figure 4:**
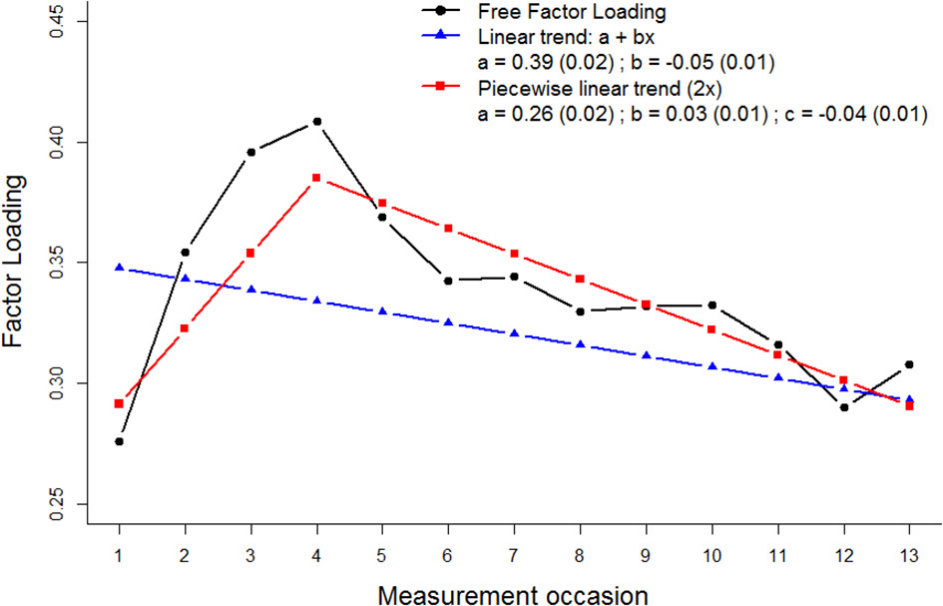
**Linear curve of measurement bias parameters of the factor loading “sickness.”** The black line (circles) represents measurement bias parameter estimates when they are freely estimated across occasions, the blue line (triangles) when they are modeled using a linear curve, and the red line (squares) represents measurement bias parameter estimates when they are modeled using two piece-wise linear curves.

This occurrence of measurement bias was modeled using a linear curve and a piece-wise linear curve (see Figure 4). The model with the linear curve showed significant deterioration in fit [CHISQ_*diff*_ (11) = 69.7, *p* < 0.001; ECVI_*diff*_ = 0.06, 95% CI: 0.02–0.12], but the model with two piece-wise curves showed equivalent fit to the model with free factor loadings [CHISQ_*diff*_ (10) = 31.1, *p* < 0.001; ECVI_*diff*_ = 0.01, 95% CI: −0.01–0.05]. The slope parameters give an indication of the steepness and direction of the measurement bias for the first three measurement occasions, and the deviations from this trend for the fourth to thirteenth measurement occasions (see Figure 4).

### Curves partial measurement invariance model

The final model, the *Curves Partial Measurement Invariance Model*, includes the three curves described above to model the measurement biases that were detected. The overall fit of the model was good (RMSEA = 0.035, see Table 1) and showed equivalent model fit when compared to the model with no curves fitted to the measurement biases [CHISQ_*diff*_ (30) = 62.5, *p* < 0.001; ECVI_*diff*_ = −0.01].

### Assessment of change

**Figure 5:**
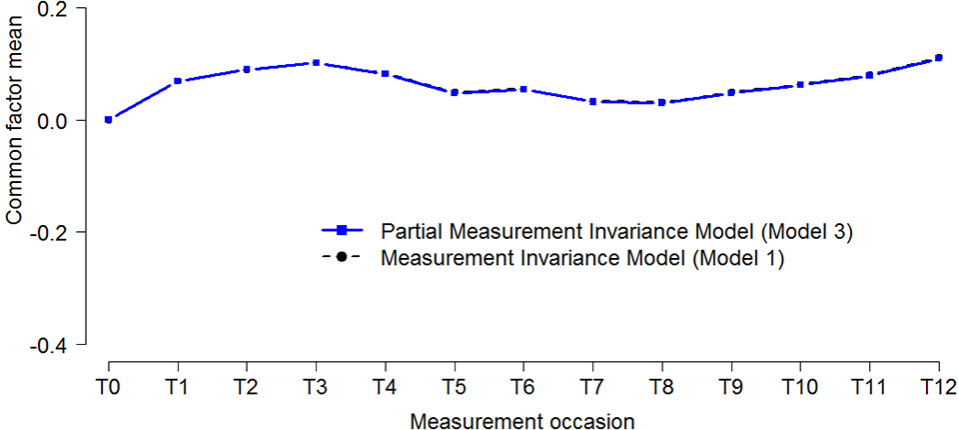
**Latent trajectories of functional limitations before and after accounting for measurement bias**. The dotted black line (circles) represents estimates of the *Measurement Invariance Model*, and the solid blue (squares) line represents parameter estimates of the *Partial Measurement Invariance Model*, where all measurement biases are incorporated in the model.

The trajectory of the common factor functional limitations (see Figure 5) indicates that patients showed a more or less constant trajectory [CHISQ_*diff*_ (12) = 39.8, *p* < 0.001; ECVI_*diff*_ = 0.02, 95% CI: −0.01–0.06]. As the biases that were detected concern the measurement of health impairments, taking into account measurement bias did not affect the trajectory of functional limitations.

**Figure 6:**
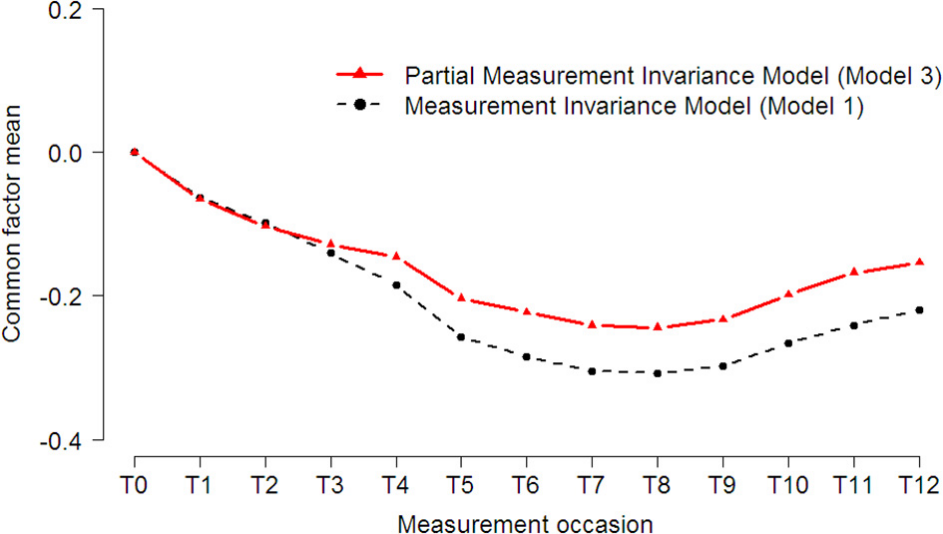
**Latent trajectories of health impairments before and after accounting for measurement bias**. The dotted black line (circles) represents estimates of the *Measurement Invariance Model*, and the solid red (triangles) line represents parameter estimates of the *Partial Measurement Invariance Model*, where all measurement biases are incorporated in the model.

The trajectory of health impairments (see Figure 6) shows that patients significantly improved [CHISQ_*diff*_ (12) = 51.5, *p* < 0.001; ECVI_*diff*_ = 0.03, 95% CI: 0.001–0.085], although it seems that patients slightly deteriorated again in the last 3 weeks of measurement. Taking into account the measurement biases of the indicators of health impairments affected the trajectory, as it can be seen that health impairments would be generally underestimated across occasions.

## Discussion

Measurement invariance is a prerequisite for a valid assessment of change. Longitudinal measurement invariance is usually investigated with a LFM. However, in the situation when there are many measurement occasions the LFM can become of unmanageable size. One solution to this problem is the imposition of Kronecker product restrictions to model the multivariate longitudinal structure of the data. In these models Kronecker product restrictions also imply measurement invariance across measurement occasions. As a result, measurement bias across occasion cannot be investigated in the usual way, by testing equality constraints on individual parameters (intercepts and factor loadings). Therefore, to investigate which measurement parameters show violations of measurement invariance (i.e., measurement bias) in Kronecker product restricted models, we propose a modeling procedure that uses additional matrices to accommodate possible bias. This enables the investigation of measurement bias, to account for apparent bias, and use partial measurement invariance to investigate change in common factor means.

The procedure that we propose enables the investigation of measurement invariance in Kronecker product restricted models for multivariate longitudinal data when the number of measurement occasions is large. The procedure of measurement invariance investigation is not different from the usual procedure, but requires alternative modeling as the usual LFM cannot be applied in the situation when invariance restrictions on factor loadings and intercepts are imposed using the Kronecker product. Moreover, with additional matrices that are used to accommodate possible violations of measurement invariance, it is possible to further investigate and model detected biases. This paper therefore contributes to the existing literature on measurement bias detection using structural equation modeling by: (1) using the imposition of Kronecker product restrictions to enable factor analyses of data from a large number of measurement occasions, (2) describing a procedure that enables measurement invariance investigation with Kronecker product restricted models, and (3) modeling the measurement bias parameters to facilitate interpretation of detected biases.

In case of bias, the detected measurement bias can be modeled as a function of the time of measurement using linear or non-linear curves. It should be noted that this technique was used in an exploratory way, e.g., the curve that was fitted to the bias was chosen after inspection of the trajectory of the measurement bias parameters. Interpretation of bias is then facilitated by decreasing the number of parameters to be interpreted, i.e., a slope parameter indicates direction and strength of the trend of the bias across time. Moreover, additional information could be used to test specific hypotheses, for example by incorporating the time of an event (e.g., start of treatment) in modeling the curves.

In our illustrative sample of bone metastases patients imposition of Kronecker product restrictions enabled the analyses of multivariate data from 13 measurement occasions, and the proposed procedure for the investigation of measurement invariance enabled the detection of measurement bias, to account for apparent bias, and use partial measurement invariance to investigate change in HRQL. We found that patients showed a constant trajectory of functional limitations and an improvement of health impairments over time. If measurement bias had not been taken into account, patient's health impairments would generally be underestimated. Moreover, measurement bias was detected in the intercept of the indicator pain, and in both the intercept and factor loading of the indicator sickness. Given equal health impairments, patients reported decreasing pain over the first 4 weeks after treatment, after which they reported stable pain over time. In addition, given equal health impairments patients reported more sickness in the first 3 weeks after treatment, after which they again reported less sickness. Similarly, the importance of sickness became more important for patients' health impairments in the first 3 weeks and then decreased again toward baseline level. A possible explanation for the bias in pain as a measurement of health impairments could be that the radiotherapy treatment led to a larger decrease in pain than in the other indicators of health impairments. In the measurement of health impairments, patients' reporting of pain would then decrease relative to the other indicators. A possible explanation for the biases in sickness could be that patients experienced side-effects from radiotherapy and that symptoms related to sickness were relatively more prevalent than the other symptoms. Sickness could therefore have become more important to the measurement of health impairments, relative to the other symptoms. As these side-effects usually disappear after a few weeks, this could explain the subsequent decrease in both the reporting of sickness relative to the other symptoms and its importance in the measurement of health impairments. These occurrences of measurement bias and their impact on the assessment of change emphasize the importance of investigating measurement invariance when analyzing longitudinal data. Our proposed procedure enables the investigation of measurement invariance in Kronecker product restricted models, and therefore allows for a more complete interpretation of findings from multivariate longitudinal data.

### Practical guidelines

The introduction of parameter matrices that can accommodate possible violations of measurement invariance enables the investigation of bias in individual factor loading and intercepts. Further investigation of cases of bias is possible through modeling the measurement bias using linear and non-linear curves. The proposed methods not only enable the investigation of measurement bias with longitudinal three-mode models, but can also enhance our understanding of occurrences of measurement bias in multivariate longitudinal data.

### Conflict of interest statement

The authors declare that the research was conducted in the absence of any commercial or financial relationships that could be construed as a potential conflict of interest.

## Footnotes

^1^OpenMx was used for statistical analyses because it provides a matrix algebraic approach to structural equation modeling that facilitates the decomposition of matrices that is required for the imposition of Kronecker product restrictions. Other statistical software (e.g., LISREL and Mplus) could also be used for statistical analyses presented in this article, but these programs require a much longer, more complicated script as they only allow inequality constraints on individual parameters.
